# Zum Verständnis von Gesundheit in der Arbeitswelt – ein Problemaufriss

**DOI:** 10.1007/s40664-022-00483-9

**Published:** 2022-10-21

**Authors:** Michael Ertel, Ute Latza, Ina Schöllgen, Ulrike Rösler, Inga Lisa Schulz, Camilla Kienast

**Affiliations:** 1 Fachbereich „Arbeit und Gesundheit“, Bundesanstalt für Arbeitsschutz und Arbeitsmedizin, Nöldnerstr. 40–42, 10317 Berlin, Deutschland; 2 Fachbereich „Arbeit und Gesundheit“, Bundesanstalt für Arbeitsschutz und Arbeitsmedizin, Fabricest. 8, 01099 Dresden, Deutschland

**Keywords:** Gesundheitsverständnis, Gesundheitsschutz in der Arbeitswelt, COVID-19, Pandemie, Soziale Ungleichheit, Understanding of health, Health protection in the world of work, COVID-19, Pandemic, Social inequality

## Abstract

Arbeitsbedingungen, die die Gesundheit und die Arbeitsfähigkeit erhalten und fördern, sind ein wesentliches Element der Prävention arbeitsbedingter Erkrankungen und ein entscheidender Faktor für die Sicherung der Teilhabe am Erwerbsleben. Die COVID-19-Pandemie und ihre weitreichenden Folgen, der beschleunigte Klimawandel und die Digitalisierung sind gewaltige Herausforderungen für die Gesundheit und den Gesundheitsschutz auch in der Arbeitswelt. Sie erfordern, über tagesaktuelle Betrachtungen hinaus, eine vertiefte Reflexion des fachlichen Verständnisses und des konzeptionellen Rahmens, die dem Begriff Gesundheit zugrunde liegen. Mit dieser Zielsetzung werden Denkanstöße für ein zeitgemäßes Verständnis von Gesundheit, zum Gesundheitsschutz in der Arbeitswelt – Prioritäten für den Schutzbedarf der Beschäftigten – und Anregungen zur diesbezüglichen Forschung zur Diskussion gestellt. Basierend auf den körperlichen, mentalen und sozialen Dimensionen von Gesundheit und deren vielfältigen Bezügen zur Arbeitswelt vermittelt der Beitrag innovative Impulse zur Entwicklung und Priorisierung von Fragestellungen für wissenschaftliche Untersuchungen zum Schutz und zur Förderung von Gesundheit im Arbeitskontext und zur Bewertung der Aussagekraft und Qualität der Ergebnisse dieser Untersuchungen für einen modernen, präventiven Arbeits- und Gesundheitsschutz, der zur menschengerechten Gestaltung von Arbeit und damit auch zur Entlastung der sozialen Sicherungssysteme beiträgt. In Übereinstimmung mit der Public-Health-Strategie für Deutschland wird soziale Ungleichheit von Gesundheit als relevantes Strukturmerkmal hervorgehoben.

## Abstract (en)

Working conditions that maintain and promote health and the ability to work are an essential element in the prevention of work-related illnesses and a decisive factor in ensuring participation in working life. The COVID-19 pandemic and its far-reaching consequences, accelerated climate change and digitalization are huge challenges for health and health protection in the world of work as well. They require a deeper reflection of the professional understanding and the conceptual framework that underlie the term health, beyond current considerations. With this objective, thought-provoking impulses for a contemporary understanding of health, for health protection in the world of work, priorities for the protection needs of employees and suggestions for related research are presented for discussion. Based on the physical, mental and social dimensions of health and their manifold relations to the world of work, the article provides innovative impulses for the development and prioritization of questions for scientific studies on the protection and promotion of health in the workplace and for evaluating the significance and quality of the results of these studies for modern preventive occupational health and safety, which contribute both to the humane design of work and to the relief of the social security systems. In accordance with the Public Health Strategy for Germany, social inequality of health is highlighted as a relevant structural feature.

## Hintergrund

Die gesellschaftlichen Herausforderungen durch die COVID-19-Pandemie, die dadurch beschleunigte Digitalisierung und insbesondere durch den Klimawandel geben Anlass, über ein zeitgemäßes Verständnis von Gesundheit im Arbeitskontext zu reflektieren. Dies erfolgt vor dem Hintergrund der zentralen Ziele des Arbeits- und Gesundheitsschutzes, die Gesundheit und Arbeitsfähigkeit der Beschäftigten zu fördern, zu erhalten und ggf. wiederherzustellen. Wie im Rahmen des Zukunftsforums Public Health definiert [30], ist Gesundheitsschutz ein Sammelbegriff für rechtlich geregelte Maßnahmen zur Abwehr von Gefahren für das Leben oder die Gesundheit der Menschen (z. B. Infektions- oder Arbeitsschutz sowie Arzneimittel- oder Lebensmittelsicherheit), die von öffentlichen Institutionen auf (Land‑)Kreis‑, Bezirks- Landes‑, Bundes- und europäischer bzw. internationaler Ebene wahrgenommen werden. Dem Gesundheitsschutz liegt anders als der Gesundheitsförderung ein pathogenetischer Handlungsansatz zugrunde [18].

### Pandemie und Gesundheit: Verstärkung sozialer Ungleichheiten

Die Pandemie ist mit massivem menschlichem Leid und erheblichen gesellschaftlichen Kosten verbunden [14]. Sie akzentuiert die Verantwortlichkeit des Staates für den Schutz von Leben und Gesundheit der Bevölkerung – dies gilt insbesondere für besonders vulnerable Gruppen – wie auch das Spannungsfeld zwischen individueller Freiheit (Selbstbestimmung) und der (abgestuften) Verantwortung der Einzelnen für das Gemeinwesen. Dass die Pandemie vor allem einen Verstärkereffekt im Hinblick auf arbeitsbezogene bzw. sozial determinierte Ungleichheit von Gesundheit hat, wurde von Siegrist aufgezeigt [23]. Sogenannte „essential workers“ oder „frontline workers“, die nicht im Homeoffice arbeiten können, waren und sind einem erhöhten Infektionsrisiko ausgesetzt, darunter verschiedene Beschäftigtengruppen mit relativ niedriger Entlohnung, z. B. Reinigungsberufe, Lagerwirtschaft, Pflegeberufe und Verkaufsberufe im Bereich Lebensmittel [16]. Hinzu kommt, dass viele Beschäftigte in diesen *systemrelevanten* Beschäftigungsgruppen mehr Gesundheitsbeeinträchtigungen haben und daher häufiger dem Risiko eines schweren Erkrankungsverlaufs ausgesetzt sind und dass die Maßnahmen zum Infektionsschutz ungleiche Folgen je nach sozioökonomischem Status haben [17]. So betreffen z. B. Einkommensausfälle wegen Kurzarbeit oder Arbeitslosigkeit verstärkt Personen in prekären Beschäftigungsverhältnissen.

In Deutschland wurden als Reaktion auf die akute Gefährdung der Bevölkerung durch lokale Corona-Hotspots in der Fleischindustrie als regulative Antwort im Arbeitsschutzkontrollgesetz [12] Maßnahmen zur Verbesserung der z. T. prekären Arbeits- und Lebensbedingungen der dort Beschäftigten und damit auch zur Verbesserung des Gesundheitsschutzes der Bevölkerung gesetzlich verankert. Dazu zählt auch das Verbot der u. a. im Hinblick auf die arbeitsmedizinische Betreuung besonders problematischen Leiharbeit in Kernbereichen der Fleischwirtschaft. Auch in weiteren Bereichen werfen die akuten Effekte der Pandemie ein Schlaglicht auf chronische gesellschaftliche Problemlagen, die staatliches Handeln erforderlich machen. Darunter fallen primär Beschäftigte im Gesundheitswesen – und hier wiederum insbesondere die in der stationären Krankenbehandlung und Pflege Tätigen – die zudem die Hauptlast der Pandemiebekämpfung tragen (für die USA [10]).

Im Hinblick auf Begleiterscheinungen und *Kollateralschäden* der Pandemiebekämpfung (wie Zusatzbelastungen durch erhöhte Anforderungen an die Alltagsbewältigung, z. B. wegen Schul- oder Kitaschließung, Verstärkung der traditionellen Geschlechterrollen bzgl. Beruf und Familie) nimmt auch die Bedeutung des Themas „Mentale Gesundheit“ zu.

### Gesundheit im Verständnis der WHO

Gerade in Zeiten globaler, z. T. miteinander verbundener und sich überlagernder Herausforderungen für die Gesundheit – durch den Klimawandel, die Pandemie, die Digitalisierung und akute globale Krisen – ist für unsere weiteren Überlegungen das umfassende Verständnis von Gesundheit bedeutsam, das in der Präambel der Konstitution der WHO verankert ist:

Health is a state of complete physical, mental and social well-being and not merely the absence of disease or infirmity. The enjoyment of the highest attainable standard of health is one of the fundamental rights of every human being without distinction of race, religion, political belief, economic or social condition. (Constitution of the World Health Organisation, 1946) [28]

Dieses werteorientierte, ethisch fundierte Gesundheitsverständnis bedarf der näheren inhaltlichen Bestimmung. Die WHO hat später bereits zwischen „Gesundheit als ein(em) Grundrecht des Menschen und als ein weltweites soziales Ziel“ und Gesundheit als „objektive(r) Messgröße“ im Sinne einer Arbeitsdefinition unterschieden [29]. Mit dieser Differenzierung wurde auch einer oft geäußerten Kritik am *utopischen* und daher für die Praxis kaum handhabbaren Charakter des ursprünglichen WHO-Gesundheitsbegriffs (z. B. [6]) Rechnung getragen. 1987 entwickelte die WHO ihre Definition im Nachgang zur 1986 verabschiedeten Ottawa Charta zur Gesundheitsförderung [27] weiter: „Gesundheit ist die Fähigkeit und die Motivation, ein wirtschaftlich und sozial aktives Leben zu führen“ [25].

### Zielsetzung

Vor dem Hintergrund der aktuellen Herausforderungen für die Gesundheit werden in diesem Beitrag Überlegungen zu einem problemadäquaten Verständnis von Gesundheit und Gesundheitsschutz in der Arbeitswelt formuliert und zur Diskussion gestellt. Da in der Pandemie wieder deutlich wurde, dass sozial ungleich verteilte Gesundheitsrisiken in einer Krisensituation akzentuiert bzw. verstärkt werden, betonen wir dabei im Einklang mit der Public-Health-Strategie für Deutschland [30] soziale Ungleichheit von Gesundheit als relevantes Strukturmerkmal [23]. Dies vorausgesetzt und vor dem Hintergrund der historisch gewachsenen, werteorientierten Begriffsbestimmungen der WHO ist insbesondere ein mehrdimensionales Verständnis von Gesundheit, wie auch eine Begründung, welche Dimensionen von Gesundheit in arbeitsweltbezogener Forschung untersucht werden sollen, ein wichtiges Qualitätsmerkmal und ein Kriterium für förderungswürdige Forschung.

Somit verfolgt dieser Beitrag zwei Ziele: Mit einer integrativen und interdisziplinären Perspektive auf wesentliche Dimensionen und Determinanten von Gesundheit wollen wir den aus unserer Sicht notwendigen konzeptionellen Rahmen für die Entwicklung und Priorisierung vertiefender, auf die Förderung und den Schutz von Gesundheit im Arbeitskontext bezogener Fragestellungen für wissenschaftliche Untersuchungen abstecken. Zugleich wollen wir Hinweise zur Beurteilung der Aussagekraft und Qualität von Forschungsergebnissen hierzu geben.

Dieser Aufgabe stellte sich ein multidisziplinäres Team aus dem Fachbereich „Arbeit und Gesundheit“ der Bundesanstalt für Arbeitsschutz und Arbeitsmedizin (BAuA). Anknüpfend an fachliche Diskurse zur begrifflichen Bestimmung von „Gesundheit“ sowie „mentaler Gesundheit“ und „psychischer Gesundheit“ in der BAuA [22, 24] wurden zunächst mehrere Workshops durchgeführt, die dazu dienten, Kernelemente eines fachlichen Selbstverständnisses von „Gesundheit“ sowie deren Bestimmungsgrößen zu erarbeiten. Hierzu wurden auf der Basis einer Zusammenschau etablierter Gesundheitsmodelle in einem mehrstufigen Verfahren in Anlehnung an die Delphi-Methode deren Kernaussagen extrahiert und diese unter dem Gesichtspunkt konsentiert, inwiefern sie für die künftige Forschung zu „Arbeit und Gesundheit“ zu berücksichtigen sind. Die innovative Qualität dieser Thesen ist in der integrativen und interdisziplinären Gesamtschau auf „Gesundheit“ in Arbeitskontext aus der Perspektive der anwendungsbezogenen Ressortforschung unter Betonung von aktuellen und sich z. T. überlagernden bzw. verschränkenden Problemlagen (Klimakrise, Pandemie, Digitalisierung, verschärfte soziale Ungleichheit) zu sehen.

## Thesen zum Verständnis von Gesundheit und zum Gesundheitsschutz

Ausgehend von der werteorientierten Definition der WHO (1946) [28] lassen sich die ersten 4 Thesen als Basis- bzw. Grundverständnis von Gesundheit festhalten, die für diesen Beitrag um 6 Thesen mit weiterführenden Überlegungen zum Arbeits- und Gesundheitsschutz (5–10) ergänzt wurden.

1. Gesundheit ist mehrdimensional. Körperliche, mentale und soziale Dimensionen sind eng miteinander verbunden.
2. Individuen müssen nicht zwingend einem dichotomen Zustand von Gesundheit oder Krankheit zugeordnet werden, sondern befinden sich auf einem dynamischen Kontinuum zwischen Gesundsein und Kranksein [24]. Die Berücksichtigung der Unterschiedlichkeit von Gesundheitsempfinden, differenziert nach Alter, Geschlecht, sozioökonomischem Status, kulturellen Faktoren und ggf. weiteren Diversitätsmerkmalen ist dabei von Bedeutung [20].
3. Gesundheit ist multifaktoriell determiniert durch Anforderungen und Ressourcen bzw. Risiko- und Schutzfaktoren. Diese Determinanten können der (Arbeits‑)Umwelt und der Person zugeordnet werden. Umwelt wird hierbei breit gefasst – dies schließt den allgemeinen Kontext der direkten Umwelt mit ein [7]. Zur Person zählen sowohl unveränderliche Eigenschaften (z. B. die genetische Ausstattung, das chronologische Alter), als auch veränderbare Eigenschaften, Fähigkeiten und Kompetenzen – Menschen sind eigenständig und zugleich in vielfältige soziale Kontexte eingebettet. Die Wirkung dieser Determinanten auf die Gesundheit ist komplex und wird bestimmt von Art, Intensität und der zeitlichen Dimension der Determinanten sowie deren Wechselwirkung.
4. Es existiert ein Spannungs- bzw. Ergänzungsverhältnis zwischen einem objektiven (auf Experteneinschätzung gestützten und diagnostizierbaren) Verständnis von Gesundheit als *Zustand* und einem subjektiven (auf Selbsteinschätzung basierenden und die individuelle Selbstbestimmung betonenden) Verständnis von Gesundheit als Handlungs*fähigkeit bzw. Kompetenz, auch bei objektiv beeinträchtigtem Gesundheitszustand *[4, 9]. Betont die Krankheitsprävention den Schutz von Individuen durch die Vermeidung bzw. Kontrolle von Risikofaktoren, liegt der Akzent der Gesundheitsförderung auf der Stärkung der Ressourcen der Individuen zur erfolgreichen Bewältigung von Belastungen [13]. Je nach Erkenntnisinteresse und dieser entsprechenden Fragestellung haben beide Perspektiven auf Gesundheit ihre Berechtigung [11].
5. „Gesundheit“ bzw. deren Beeinträchtigung ist in unterschiedlicher Weise sichtbar. Der daraus resultierende Schutzbedarf unterscheidet sich im Hinblick auf dessen wahrgenommene Dringlichkeit. So hat die – temporär notwendige – Fokussierung der Aufmerksamkeit auf die Infektionsgefährdung in der Pandemie auch eine problematische Seite: Risikowahrnehmung und Problembearbeitung orientieren sich an der *„drastische(n) Sichtbarkeit schwerer, stets akuter Verläufe dieser neuen Infektion und (den) daraus für die Allgemeinheit klar ersichtlichen Gefahren und Schutzmaßnahmen“* [23, S. 210]. Dem steht die vergleichsweise *„Unsichtbarkeit sozial ungleicher Morbidität und Mortalität, die langen Zeiträume ihrer Entstehung und die Unklarheit bezüglich der zu ergreifenden Gegenmaßnahmen“* [23, S. 210] gegenüber. Daher ist eine Schärfung der Forschungsperspektiven im Hinblick auf die Wahrnehmung gesellschaftlicher Risiken im Gesamt-Zusammenhang erforderlich.
6. Durch die Zunahme mobiler Arbeit in der Pandemie kommt der psychosozialen Dimension bzw. der psychischen Gesundheit eine hohe und weiter zunehmende Bedeutung für den Arbeitskontext zu [19]. *Beeinträchtigte psychische Gesundheit* in der Arbeitswelt verdient auch aufgrund des erhöhten Risikos einer Chronifizierung und der damit verbunden Langzeitarbeitsunfähigkeit aufgrund von psychischen Erkrankungen bis hin zur Frühberentung sowie der Probleme durch Unterschätzung der Häufigkeit („underreporting“) und Präsentismus besondere Aufmerksamkeit [24].
7. Der durch die Pandemie ausgelöste bzw. verstärkte Digitalisierungsschub, abzulesen insbesondere an der starken Zunahme von Onlinekommunikation (Videokonferenzen) und der Ausbreitung internetbasierter Geschäftsmodelle unter dem Stichwort der Plattform-Ökonomie, hat zugleich die Aufmerksamkeit auf Defizite von Infrastrukturen, Arbeits‑, Planungs- und Verwaltungsprozessen gelenkt. Der forcierte Ausbau der digitalen Infrastruktur stellt daher eine prioritäre gesellschaftliche Aufgabe dar [15]. Begleitend ist eine integrierte Potenzial- und Risikobetrachtung erforderlich, welche Digitalisierung als Ressource und zugleich – aufgrund der Ambivalenz digitaler Techniken und Prozesse – als möglichen Risikofaktor für die Gesundheit in den Blick nimmt [2].
8. Der beschleunigte Klimawandel stellt eine massive globale Bedrohung dar [15] und ist für die Arbeitswelt aus gesundheitlicher Perspektive auch jenseits etablierter Schwerpunkte des Arbeitsschutzes (z. B. Hitzearbeitsplätze im Produktionsbereich und Arbeitstätigkeiten im Außenbereich) von Bedeutung. Dies betrifft z. B. die Auswirkungen von erhöhten Temperaturen auf Psyche und Leistungsfähigkeit wie auch notwendige Anpassungsprozesse von Arbeitsprozessen, Arbeitsumgebung und Arbeitsorganisation an den Klimawandel. Zudem gilt das Thema „Klimaschutz für Beschäftigte“ als „blinder Fleck“ [1] im Arbeits- und Gesundheitsschutz und bedarf daher der gezielten Bearbeitung [8], auch unter dem Aspekt der globalen Gerechtigkeit [5].
9. Aus einer Public-Health-Perspektive und im globalen Maßstab relevant sind insbesondere ein sozial gerechter Zugang zur Gesundheitsversorgung sowie ein freier Zugang zu Gesundheitsinformationen [23, 30].
10. Perspektivisch sollte das Thema Gesundheit stärker Disziplinen übergreifend bzw. transdisziplinär betrachtet und bearbeitet werden. Transdisziplinarität umfasst innerwissenschaftliche Zusammenarbeit und den Diskurs zwischen Forschung und Gesellschaft. Probleme sowie Forschungsfragen können eher erfolgreich bearbeitet werden, wenn diese aus verschiedenen Perspektiven betrachtet und partizipativ untersucht werden [3, 21].

## Diskussion

Arbeitsbedingungen, die die Gesundheit und die Arbeitsfähigkeit erhalten und fördern, sind ein wesentliches Element der Prävention arbeitsbedingter Erkrankungen und ein entscheidender Faktor für die Sicherung der Teilhabe am Erwerbsleben. Für einen modernen präventiven Arbeits- und Gesundheitsschutz, der Präventionspotenziale umfassend ausschöpft, sind die körperlichen, mentalen und sozialen Dimensionen von Gesundheit und ihre multifaktoriellen Determinanten aus Person und (Arbeits‑)Umwelt interdisziplinär zu betrachten, um das dynamische Kontinuum zwischen Gesundheit und Krankheit in Richtung des Erhalts und der Förderung von Gesundheit zu verschieben [2].

Aus unserer Diskussion des Gesundheitsbegriffs bzw. des Gesundheitsverständnisses sollte dessen Facettenreichtum und begründete Vielfalt (nicht Beliebigkeit) deutlich geworden sein. Je nach Frage- und Problemstellung in den Bereichen von Forschung, Politikberatung und Praxistransfer in der Arbeitswelt stehen unterschiedliche Dimensionen von Gesundheit (z. B. subjektives Wohlbefinden, körperliche Funktionsfähigkeit, Störungsfreiheit, Leistungsfähigkeit) im Vordergrund. Die problem- und anwendungsbezogene Forschung – insbesondere die Ressortforschung – sollte den verschiedenen Akteurinnen und Akteuren des Arbeits- und Gesundheitsschutzes, der Gesundheitspolitik und anderer Politikbereiche eine evidenzbasierte Entscheidungsgrundlage zum Erhalt und zur Förderung von Gesundheit in ihren Bereichen und ressortübergreifend zur Verfügung stellen.

Mit der hier skizzierten integrativen und interdisziplinären Perspektive auf wesentliche Dimensionen und Determinanten von Gesundheit können differenzierte Kriterien für die Planung und Bewertung von Forschungsansätzen zur Förderung und den Schutz von Gesundheit im Arbeitskontext entwickelt werden. Dabei muss der Vielschichtigkeit von Gesundheit und ihrer Determinanten in der Arbeitswelt sowohl in den Forschungsansätzen als auch in der Bewertung der Aussagekraft ihrer Ergebnisse Rechnung getragen werden. Hierfür ist es erforderlich, dass die Forschenden transparent machen, welche Dimensionen und Determinanten von Gesundheit bzw. Indikatoren aufgrund welcher theoretischen Vorüberlegungen betrachtet werden, wie umfassend diese Betrachtung ist und welche Aussagen für den Arbeits- und Gesundheitsschutz davon abgeleitet werden können. Konzeptionell fundierte und zugleich praxisnahe Fragestellungen erfordern jeweils eine mehrdimensionale Betrachtung einschließlich der damit ggf. verbundenen Ambivalenzen bzw. Abwägung von unterschiedlichen Interessenlagen, Rahmenbedingungen, subjektiven Perspektiven und Zeithorizonten. So kann beispielsweise eine komprimierte Arbeitswoche mit längeren Arbeitszeiten an weniger Arbeitstagen aus Sicht der Beschäftigten zunächst die Work-Life-Balance und/oder die selbstberichtete Gesundheit positiv beeinflussen und daher präferiert werden, aber gleichzeitig – aus arbeitsmedizinischer bzw. arbeitsschutzbezogener Perspektive – die Unfallgefahr und langfristig das kardiometabolische Risiko erhöhen. Komplexe Problemlagen mit umfassenden und weitreichenden Konsequenzen, insbesondere der Klimawandel und dessen Auswirkungen auf die Gesundheit (am Arbeitsplatz), erfordern breit angelegte Konzepte und ein Zusammenwirken von Public Health und Arbeits- und Gesundheitsschutz („occupational health“). Dabei sind arbeitsbezogene und durch Maßnahmen des Arbeits- und Gesundheitsschutzes (positiv) beeinflussbare Determinanten – deren Priorisierung und deren Zusammenwirken – wesentlich, wobei auch personenbezogene Faktoren – z. B. Gender – im Rahmen der Forschungsmethodik und des Arbeits- und Gesundheitsschutzes berücksichtigt werden müssen. So ist z. B. in der Pandemie zu berücksichtigen, dass traditionelle Geschlechterrollen bzgl. Beruf und Familie generell verstärkt wurden. Als zentrale Querschnittsdimension sollte jeweils auch der Stellenwert sozialer Ungleichheit von Gesundheit (z. B. Gesundheitsrisiken wie Gesundheitschancen von prekär beschäftigten Personen) reflektiert und untersucht werden, wie diese in der Arbeitswelt erkannt, abgebaut bzw. positiv beeinflusst werden kann [26].

Die geforderte Transparenz und bewusste Auseinandersetzung mit den Begriffen „Gesundheit“ und „Gesundheitsschutz“ sollen einerseits zu Respekt gegenüber unterschiedlichen Forschungsansätzen beitragen. Andererseits sollen Impulse vermittelt werden, um handlungsleitende Fragestellungen für die Weiterentwicklung des Arbeits- und Gesundheitsschutzes abzuleiten, zu untersuchen und so zu einer menschengerechten Gestaltung von Arbeit und einer Entlastung der sozialen Sicherungssysteme beizutragen.
